# From research to impact: two decades of advancing age-friendly policies worldwide

**DOI:** 10.1093/ppar/prag010

**Published:** 2026-05-09

**Authors:** Jarmin Yeh, James D. Harrison, Sunny C. Lin, Jade Christey, Julia Adler-Milstein

**Affiliations:** 1 Department of Social and Behavioral Sciences, Institute for Health & Aging, University of California, San Francisco, California, United States; 2 Division of Hospital Medicine, University of California, San Francisco, California, United States; 3 School of Medicine, Washington University, Saint Louis, Missouri, United States; 4 Division of Clinical Informatics & Digital Transformation, University of California, San Francisco, California, United States; 5 Division of Clinical Informatics & Digital Transformation, University of California, San Francisco, California, United States

**Keywords:** Age-friendly, Implementation science, Improvement science, Health equity

At the 2005 International Association of Gerontology and Geriatrics (IAGG) World Congress in Rio de Janeiro, a transformative concept emerged: cities could be designed to be age-friendly, supporting older adults’ health, participation, and security (WHO, 2007). This catalyzed a global movement reshaping how population aging is addressed, with initiatives spanning clinical care, communities, public health systems, and rapidly rising academic publications (Figure 1).

This Policy Spotlight examines how research has shaped the evolution and implementation of age-friendly initiatives. We highlight four complementary frameworks—the eight domains of age-friendly cities and communities (WHO, 2007), 4Ms of age-friendly health systems (Mate et al., 2018), 6Cs of age-friendly public health systems (De Biasi et al., 2020), and six characteristics of age-friendly ecosystems (Fulmer et al., 2023)—demonstrating how evidence drives policy impact across clinical, community, and system levels, while identifying gaps and challenges in supporting diverse older adults worldwide.

## Evolution of age-friendly frameworks

### Eight Domains: Age-friendly cities and communities

Following the 2005 IAGG World Congress, the World Health Organization (WHO) (2007)led a participatory research project across 33 cities, using focus groups with older adults, caregivers, and service providers to develop the age-friendly cities framework. This identified eight interconnected domains: outdoor spaces and buildings, transportation, housing, social participation, respect and social inclusion, civic participation and employment, communication and information, and community support and health services. Research stemming from this framework has centered older adults’ lived experiences and used community-level outcomes as indicators of age-friendliness (Greenfield & Buffel, 2022).

Over 1,700 cities and communities in 57 countries now implement, evaluate, and share learnings about the eight domains through WHO’s Global Network of Age-Friendly Cities and Communities. However, evaluation methods and indicators inadequately address older adults’ needs during life transitions and societal changes (Xu et al., 2025). Evidence-based refinement is needed to improve measurement tools addressing equity and structural barriers, particularly for rural areas, marginalized populations, and older adults with disabilities or cognitive impairments.

### 4Ms: Age-friendly health systems

While the WHO’s framework emphasized community environments, parallel research identified the need for age-specific clinical models across healthcare settings, including hospitals, emergency departments, nursing homes, ambulatory care, and home health. In 2016, the John A. Hartford Foundation (JAHF) and Institute for Healthcare Improvement (IHI) introduced the 4Ms framework—What Matters, Medication, Mentation, and Mobility—based on evidence that goal-aligned care, medication reviews, cognitive health monitoring, and fall prevention improves outcomes and independence for older adults (Mate et al., 2018).

Over 5,500 health systems have now committed to implementing the 4Ms, with 2,500 reporting data on older adults receiving 4Ms care. The Centers for Medicare and Medicaid Services (CMS) further sanctioned age-friendly principles by adopting the Hospital Inpatient Quality Reporting (IQR) Measure in 2025, paying hospitals to report on 4Ms care practices alongside patient social vulnerability and leadership commitment (CMS, 2024). This policy represents direct translation of an evidence-based care model into federal regulatory requirements (Adler-Milstein et al., 2025). However, challenges remain in measurement fidelity and generating evidence that 4Ms implementation improves patient-centered outcomes beyond process compliance (Burke & Pelton, 2025; Harrison et al., 2025).

### 6Cs: Age-friendly public health systems

Recognizing that clinical care and community environments alone cannot fully address older adults’ needs, the Trust for America’s Health and JAHF developed the 6Cs framework in 2017 to support public health. Grounded in research on effective community-based initiatives, the 6Cs—Creating policy change, Connecting multi-sector partners, Coordinating services, Collecting data, Communicating information, and Complementing existing programs—provide systems-level infrastructure to bridge clinical and community efforts (De Biasi et al., 2020). For example, if a hospital using the 4Ms identifies a patient’s mobility challenges stem from inaccessible transportation to physical therapy, the 6Cs framework facilitates cross-sector collaboration to improve care transitions and continuity (Wolfe & Gracia, 2023).

### Six Characteristics: Age-friendly ecosystems

The concept of age-friendly ecosystems emerged to unify fragmented efforts across health systems, communities, universities, public health, and employers (Fulmer et al., 2025). In 2020, the JAHF and Age-Friendly Institute convened research and policy experts who identified six characteristics: responsive, equitable, engaging, healthful, active, and respectful. These characteristics emphasize shared language, inclusivity, and measurable collective impact to address diverse needs, combat ageism, and empower older adults. This coordinated, evidence-based approach aims to improve older adults’ quality of life by aligning cross-sector efforts and integrating policies and practices globally (Fulmer et al., 2023).

## Research-to-policy pathways

Age-friendly research employs diverse, multidisciplinary methods. Engagement science and participatory approaches harness the lived experiences of older adults to inform interventions, implementation science uses Plan-Do-Study-Act cycles for rapid quality improvement, and electronic health records enable pragmatic evaluation of 4Ms outcomes. University research centers play critical roles in synthesizing data, fostering multi-stakeholder collaborations, and building learning health systems.

Research has effectively translated age-friendly concepts into actionable policies. For instance, 40% of Americans live in communities enrolled in AARP’s Network of Age-Friendly States and Communities, an affiliate of the WHO’s Global Network that supports US communities in implementing and assessing initiatives across the eight domains (AARP, 2025). California’s Master Plan for Aging incorporates evidence-informed age-friendly principles into housing, healthcare, economic security, caregiving, and inclusion initiatives, while Florida’s age-friendly health system pilot embedded healthy aging objectives into the state’s health improvement plan (De Biasi et al., 2020; Miller, 2021). Federal initiatives, like CMS’s age-friendly hospital IQR measure and the widespread adoption of 4Ms care at Veterans Affairs facilities, highlight the national impact of evidence-informed policies (CMS, 2024; Schwartz et al., 2025). Globally, cities like Ottawa, Manchester, and Akita adapted the WHO’s age-friendly framework to local needs, demonstrating its scalability (Rémillard-Boilard et al., 2020). Additionally, IHI’s “action communities” accelerate 4Ms implementation in healthcare settings through academic partnerships and cross-continental learning, signaling growing global commitment to evidence-based, sustainable aging policies.

## Building evidence-informed age-friendly ecosystems

Two decades after the IAGG World Congress, age-friendly research has informed policy through age-friendly frameworks including the eight domains for communities, 4Ms for clinical care, 6Cs for public health, and six characteristics for ecosystems (De Biasi et al., 2020; Fulmer et al., 2023, 2025; Mate et al., 2018; World Health Organization, 2007). Despite tremendous progress, equity gaps persist as older adult populations are increasingly diverse across race/ethnicity, language, immigration status, sexual orientation, gender identity, disability, living arrangement, and socioeconomic status. Rural communities face compounded challenges from isolation, workforce shortages, and limited municipal capacity. Climate change demands greater attention to age-friendly emergency preparedness and community resilience. Current priorities include strengthening outcomes evidence, sustaining implementation, embedding equity, and accelerating evidence-to-policy translation through multi-stakeholder collaboration to create responsive ecosystems that support diverse older adults worldwide.

## Figures and Tables

**Figure 1:**
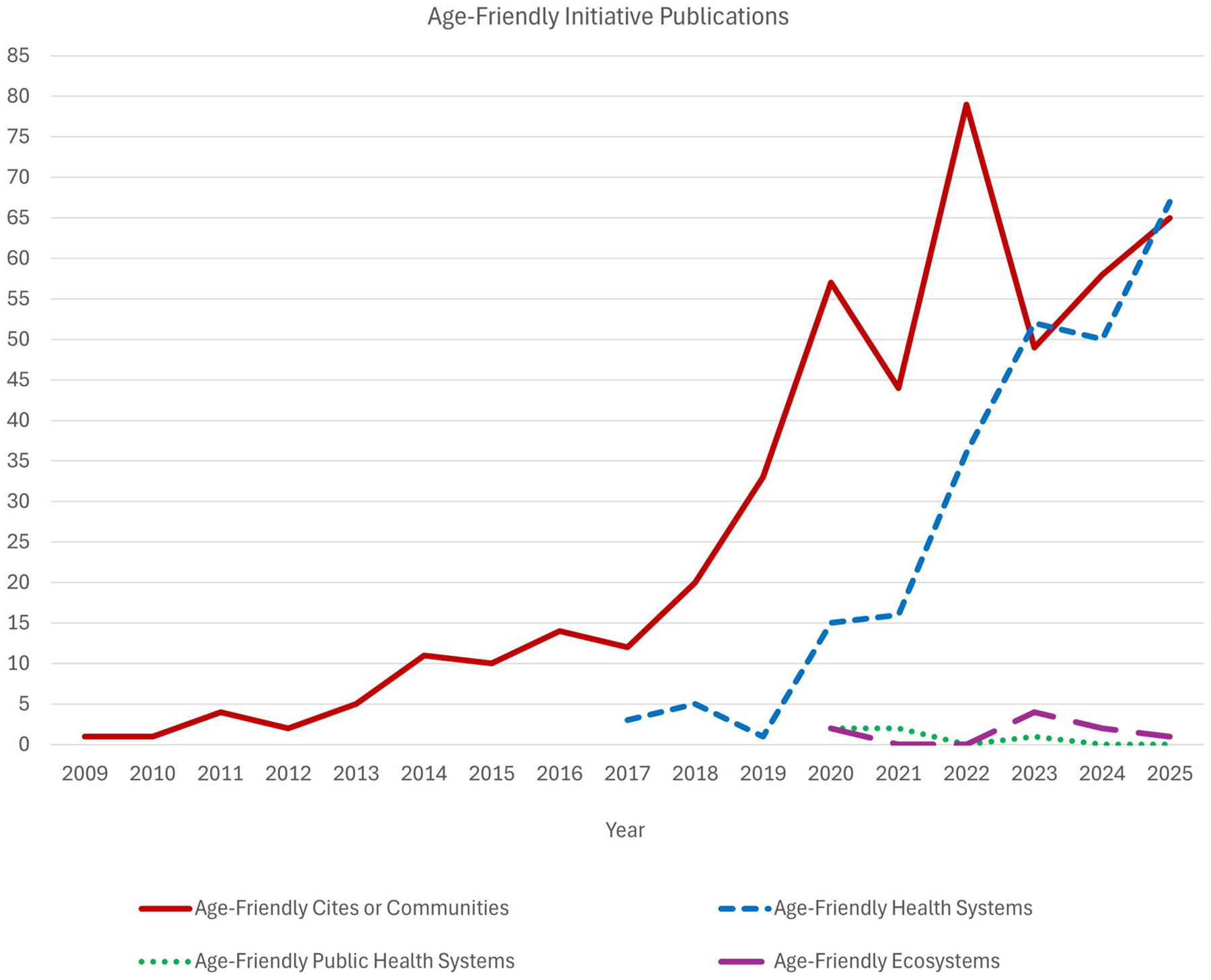
Number of academic publications regarding age-friendly initiatives. Publications with (“Age-Friendly Cit*”[Title/Abstract]) OR (“Age-Friendly Communit*”[Title/Abstract]), “Age-Friendly Health System*”[Title/Abstract], “Age-Friendly Public Health System*”[Title/Abstract], and “Age-Friendly Ecosystem*”[Title/Abstract] were searched in PubMed to estimate the number of annual publications per year. An asterisk (*) creates a wildcard search in PubMed to broaden results.

## Box

Two decades after the 2005 IAGG World Congress, age-friendly research has informed policy through frameworks including the eight domains for communities, 4Ms for clinical care, 6Cs for public health, and six characteristics for ecosystems.

Current priorities include strengthening outcomes evidence, sustaining implementation, embedding equity, and accelerating evidence-to-policy translation through multistakeholder collaboration to create responsive ecosystems that support diverse older adults worldwide.
